# Mutational analysis of primary central nervous system lymphoma

**DOI:** 10.18632/oncotarget.2080

**Published:** 2014-06-08

**Authors:** Aurélie Bruno, Blandine Boisselier, Karim Labreche, Yannick Marie, Marc Polivka, Anne Jouvet, Clovis Adam, Dominique Figarella-Branger, Catherine Miquel, Sandrine Eimer, Caroline Houillier, Carole Soussain, Karima Mokhtari, Romain Daveau, Khê Hoang-Xuan

**Affiliations:** ^1^ Sorbonne Universités, UPMC Univ Paris 06, UM 75, ICM, F-75013 Paris, France; ^2^ Institut National de la Santé et de la Recherche Médicale, U1127, ICM, Paris, F-75013 Paris, France; ^3^ Centre National de la Recherche Scientifique, UMR 7225, ICM, Paris, F-75013 Paris, France; ^4^ ICM, Paris, 75013 France; ^5^ Plateforme de Génotypage Séquençage, ICM, F-75013, Paris, France; ^6^ Onconeurothèque, Groupe Hospitalier Pitié-Salpêtrière, Assistance Publique-Hôpitaux de Paris, Paris, France; ^7^ Centre Hospitalier Universitaire Lariboisière, Assistance Publique-Hôpitaux de Paris, Service d'Anatomopathologie, Paris, France; ^8^ Hospices Civils de Lyon, Hôpital Neurologique, Bron, France and Université Lyon 1, Institut National de la Santé et de la Recherche Médicale Unité 842, Lyon, France; ^9^ Centre Hospitalier Universitaire Bicêtre, Assistance Publique-Hôpitaux de Paris, Service d'anatomopathologie, Bicêtre, France; ^10^ Centre Hospitalier Universitaire La Timone, Assistance Publique-Hôpitaux de Marseille, Institut National de la Santé et de la Recherche Médicale Unité 911, Centre de Recherches en Oncologie biologique et Onco-pharmacologie, Université de la Méditerranée and Tumorothèque de l'Assistance Publique-Hôpitaux de Marseille (AC 2013-1786), Marseille, France; ^11^ Centre hospitalier Sainte Anne, Université Paris Descartes, Sorbonne Paris Cité, Paris, France; ^12^ Service de Pathologie, CRB Tumorothèque, Centre Hospitalier Universitaire Bordeaux, Bordeaux, France; ^13^ Assistance Publique-Hôpitaux de Paris, Hôpital de la Pitié-Salpêtrière, Service de Neurologie 2-Mazarin, Paris, France; and the LOC network (INCa); ^14^ Hôpital René Huguenin, Institut Curie, Service d'Hématologie, Saint Cloud, France; and the LOC network (INCa); ^15^ Institut National de la Santé et de la Recherche Médicale Unité 830, Génétique et Biologie des Cancers, Institut Curie, Paris, France

**Keywords:** Primary CNS lymphoma, exome sequencing, somatic mutations, NFΚB, B cell differentiation

## Abstract

Little is known about the genomic basis of primary central nervous system lymphoma (PCNSL) tumorigenesis. To investigate the mutational profile of PCNSL, we analyzed nine paired tumor and germline DNA samples from PCNSL patients by high throughput exome sequencing. Eight genes of interest have been further investigated by focused resequencing in 28 additional PCNSL tumors to better estimate their incidence. Our study identified recurrent somatic mutations in 37 genes, some involved in key signaling pathways such as NFKB, B cell differentiation and cell cycle control. Focused resequencing in the larger cohort revealed high mutation rates for genes already described as mutated in PCNSL such as *MYD88* (38%), *CD79B* (30%), *PIM1* (22%) and *TBL1XR1* (19%) and for genes not previously reported to be involved in PCNSL tumorigenesis such as *ETV6* (16%), *IRF4* (14%), *IRF2BP2* (11%) and *EBF1* (11%). Of note, only 3 somatically acquired SNVs were annotated in the COSMIC database. Our results demonstrate a high genetic heterogeneity of PCNSL and mutational pattern similarities with extracerebral diffuse large B cell lymphomas, particularly of the activated B-cell (ABC) subtype, suggesting shared underlying biological mechanisms. The present study provides new insights into the mutational profile of PCNSL and potential targets for therapeutic strategies.

## INTRODUCTION

Primary central nervous system lymphoma (PCNSL) represents a rare subgroup of diffuse large B-cell lymphoma (DLBCL) that arises in the brain, eyes, meninges or spinal cord, accounting for up to 5% of primary malignant brain tumors and 1% of non-Hodgkin's lymphomas (NHL) in adults. Despite the application of intensive treatment including high-dose methotrexate based poly-chemotherapy with or without whole brain radiotherapy, the median overall survival ranges from 2 to 4 years with a poorer prognosis than extracerebral DLBCL [1]. The pathogenesis of PCNSL remains largely unclear, which is partly due to the rarity of the tumor tissue available for research studies. Transcriptomic studies have identified deregulated genes involved in the IL4/JAK/STAT6, cell adhesion-related, unfolded protein response (UPR) and apoptosis signaling pathways [2-5]. Copy number variation studies [4, 6-8] have revealed frequent chromosome losses affecting the 6q, 6p21.32 and 9p21 regions. However, the mutational landscape of PCNSL is still poorly known. A whole exome sequencing strategy has successfully identified pivotal gene mutations in several hematologic and brain malignancies [9, 10]. In a previous study, we have reported preliminary results based on four PCNSL cases investigated by this technique and identified recurrent mutations in *MYD88* and *TBL1XR1* [8]. Here, we have expanded our series and we present the results of nine paired germline and tumour samples, allowing for the identification of recurrent gene mutations that have not yet been reported in PCNSL. We confirmed the most relevant mutations and genes in a validation set of 28 PCNSL cases.

## RESULTS

### Mutational pattern of PCNSL revealed by whole exome sequencing

**Figure 1:**
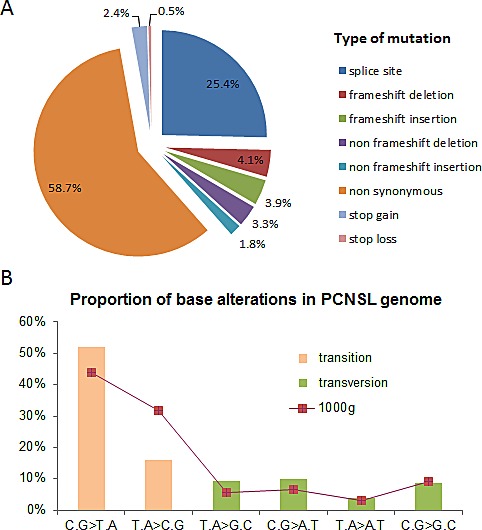
Mutation pattern of PCNSL samples investigated by whole exome sequencing To address PCNSL mutational profile, whole exome sequencing was conducted in nine paired blood and tumor samples. (A) Pie chart represents relative distribution of somatically acquired mutations classified according to their type. (B) Histogram depicts the proportion of PCNSL somatic mutations in each mutational class of transitions and transversions compared with 1000-genomes project data (red line).

To investigate the mutational profile of PCNSL, we performed high throughput exome sequencing on 9 cases. DNA from case-matched blood was also sequenced to screen out germline polymorphisms. On average, 9.8e7 (8.1e7-1.4e8) 75-bp paired reads were sequenced per sample, 5.8e7 (3.8e7-8.3e7) of these were specifically positioned onto the human reference exome (as defined by the Agilent SureSelect 50 Mb probes) after the removal of both low-quality mapped reads and potential PCR-derived duplicates (Supplementary Figure 1). This provided 76% (64-86) coverage over the targeted regions at a minimum depth of 20X (Supplementary Figure 2), wherein 82% of the bases were suitable for variant detection. Across the coding regions of the 9 matched tumor and germline pairs we investigated, we detected 17e3 (15e3-19e3) SNVs and 226 (176-263) indels. A total of 25e3 (20e3-32e3) SNVs and 21e2 (18e2-27e2) indels were also called outside of the targeted exons, but those primarily fell into neighboring introns and, in most cases, were already described as known polymorphisms. To assess the quality of our calls, we reviewed population-scale variant distributions from the 1000-genomes project and found no difference with either paired germline or PCNSL samples when considering all high-quality called SNVs and comparing the (i) transition to transversion rates, (ii) mutational spectrum and (iii) variant annotation (Supplementary Figure 3). Then, we focused on somatic SNVs identified in tumor DNA and not present in germline DNA (Figure 1). On average, we identified 220 (126-358) somatically acquired point mutations per sample and no hypermutated tumors were found. Among them, 62 (26-101) and 143 (89-231) were synonymous and non-synonymous, respectively. The non-synonymous to synonymous ratio was thus 2.4 (1.8-3.4), and there was a non-silent mutation rate of 2.9 (1.8-4.6) per Mb, the latter being lower than previously published estimations in DLBCL [11]. Half of those non-synonymous SNVs, i.e., 74 (49-111) were predicted as functionally deleterious in the dbNSFP database [12, 13]. Transitions accounted for 68% of somatic events (Figure 1B) similar to pattern observed in DLBCL [14, 15]. To confirm the depth at the somatic mutation sites, reads aligned at these genomic positions were visualized using IGV software (Broad Institute).

### Identification of 37 genes recurrently affected by somatic non-synonymous mutations

**Figure 2:**
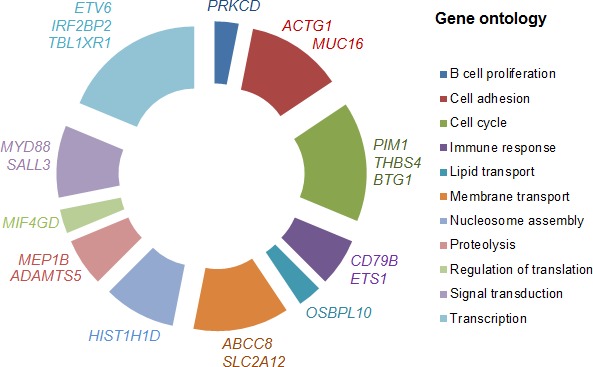
Gene ontology of PCNSL genes Relative distribution of the 37 genes somatically mutated in PCNSL by gene ontology categories. The spans of the arcs indicate the relative numbers of genes annotated with respect to gene ontology terms. Representative genes in each category are shown next to each arc.

Only SNVs located within coding regions were considered. After having removed germline variations, synonymous SNVs, indels and known polymorphisms, we identified 37 genes, harboring 142 somatically point mutations (Supplementary Table 1), that were mutated in at least 2 patients. Among these 142 mutations, 133 led to an amino acid exchange while the remaining nine led to the gain or loss of a stop codon. These 37 recurrently mutated genes were prioritized based on (i) the number of mutated tumors, (ii) the prediction of the functional impact and (iii) the number of SNVs per gene (Table 1). Then, somatic mutations were verified by Sanger sequencing on tumor and germline DNA. For *PIM1* and *MYD88* genes, only “hot spot” mutations E226K and L265P, respectively, were validated. To better understand the biological processes that are potentially altered by somatic mutations, we used gene ontology [16] annotations for these 37 genes. This functional categorization highlighted the variability of the biological processes that are altered in PCNSL (Figure 2), including transcription (e.g., *ETV6*, *IRF2BP2*, *EBF1*, *IRF4*, *TBL1XR1*), cell cycle (e.g., *PIM1*, *BTG1*), nucleosome assembly (e.g., *HIST1H1D*, *HIST1H2AC*) and cell adhesion (e.g., *MUC16*, *ACTG1*). In terms of signaling pathways, we identified mutations in the genes involved in the NFKB, WNT and B-cell or T-cell receptor signaling pathways.

**Table 1:** Prioritization of the 37 genes of interest identified by whole exome sequencing The present study identified 37 genes affected by non-synonymous somatic SNVs in at least 2 of the 9 patients of the discovery set. In this table are listed all these genes prioritized according to (i) number of mutated patients, (ii) functional impact prediction (FISM), (iii) number of mutations per gene. Functional impact prediction columns indicate the number of patients harboring at least one mutation for each FISM category (1 corresponds to the highest impact). Of importance, number of mutated patients and number of mutations per gene take into account all somatic mutations identified by exome sequencing before any attempt of validation.

				Functional prediction impact (FISM)						
Genes	Chromosome	Mutations	Patients	NA	≥0.5	≥0.6	≥0.7	≥0.8	≥0.9	=1
*PIM1*	6	32	8	0	8	8	7	6	6	5
*IGLL5*	22	12	6	6	0	0	0	0	0	0
*MYD88*	3	2	5	5	0	0	0	0	0	0
*TBL1XR1*	3	4	4	0	4	4	4	4	4	3
*CSMD3*	8	4	4	0	4	4	4	3	3	1
*CD79B*	17	3	3	0	2	2	2	2	2	1
*HIST1H2AC*	6	8	3	0	3	3	3	3	1	1
*ETV6*	12	5	3	0	3	3	2	2	1	1
*KLHL14*	18	7	2	0	2	2	2	2	2	2
*IRF4*	6	3	2	0	2	2	2	2	2	2
*PRKCD*	3	2	2	0	2	2	2	2	2	2
*ABCC8*	11	2	2	0	2	2	2	2	2	1
*ZFHX4*	8	2	2	0	2	2	2	2	2	1
*SALL3*	18	2	2	0	2	2	2	1	1	1
*IRF2BP2*	1	3	2	0	2	2	1	1	1	1
*CD37*	19	2	2	0	2	2	1	1	1	1
*OSBPL10*	3	7	2	0	2	2	2	2	2	0
*EBF1*	5	3	2	0	2	2	2	2	2	0
*DST*	6	2	2	0	2	2	2	2	1	0
*MIF4GD*	17	2	2	0	2	2	2	2	1	0
*HIST1H1D*	6	3	2	0	2	2	2	1	1	0
*BTG1*	12	2	2	0	2	2	2	1	1	0
*MEP1B*	18	2	2	0	2	2	2	1	1	0
*THBS4*	5	2	2	0	2	2	2	1	1	0
*ADAMTS5*	21	2	2	0	2	2	1	1	1	0
*HIST1H1E*	6	2	2	0	2	1	1	1	1	0
*MPEG1*	11	3	2	1	1	1	1	1	1	0
*OBSCN*	1	2	2	0	2	2	2	2	0	0
*C10orf71*	10	2	2	0	2	2	2	1	0	0
*HMCN1*	1	2	2	0	2	2	2	1	0	0
*MYH4*	17	2	2	0	2	2	1	1	0	0
*TBC1D4*	13	2	2	0	2	1	1	1	0	0
*SLC2A12*	6	2	2	0	2	2	1	0	0	0
*ETS1*	11	2	2	0	2	2	0	0	0	0
*MUC16*	19	2	2	2	0	0	0	0	0	0
*UNC80*	2	2	2	2	0	0	0	0	0	0
*ACTG1*	17	1	2	2	0	0	0	0	0	0

### Analysis of 8 relevant genes in an independent series of 28 PCNSL

**Figure 3:**
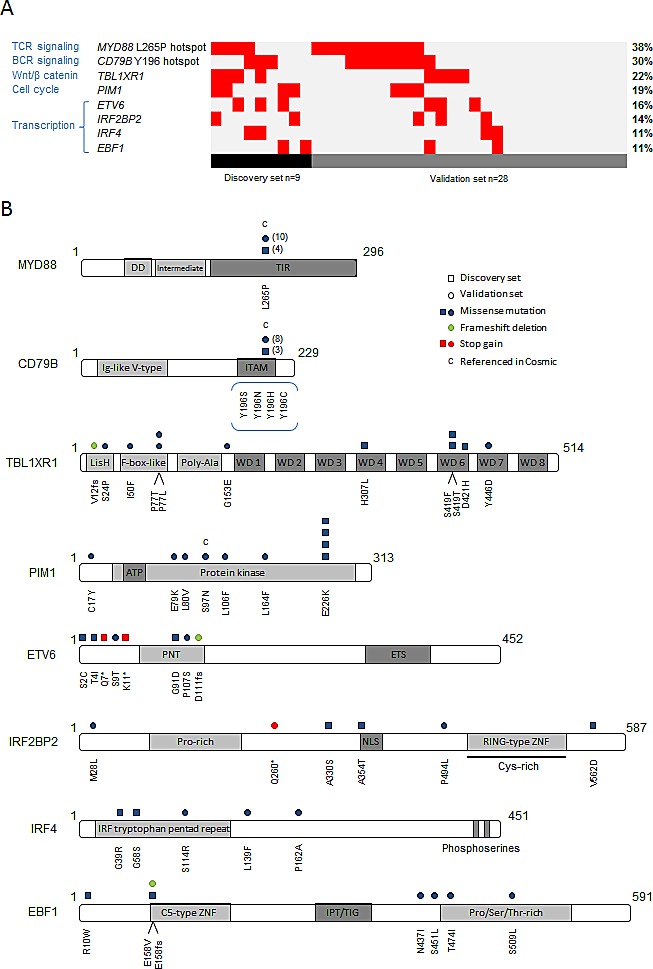
Investigation of 8 relevant genes recurrently affected by point mutations in PCNSL Based on genes identified by whole exome sequencing, we selected 8 relevant genes to be sequenced in a larger cohort: *CD79B*, *EBF1*, *ETV6*, *IRF4*, *IRF2BP2*, *MYD88*, *PIM1* and *TBL1XR1*. (A) Repartition of validated mutations by gene within the whole population of 37 PCNSL cases. (B) Schematic representation of all validated mutations identified in the discovery (□) and the validation sets (○) with their position according to protein domains. Symbol color indicates mutation type. Number of □ or ○ indicates the number of mutated patients except for L265P *MYD88* and Y196 *CD79B* mutations.

In order to specify their mutation frequency in PCNSL, we selected 8 genes for further investigation in an independent validation panel of PCNSL tumors (n=28). This selection was based both on high mutation rate in our discovery set and biological relevance. *PIM1*, *TBL1XR1*, *ETV6*, *IRF4*, *IRF2BP2* and *EBF1* were resequenced for their coding exons by pyrosequencing. We identified 133 variations, including 122 SNVs and 11 deletions. Among them, 39 variations were missense mutations (Supplementary Table 2), including 35 variations that were not previously described in the dbSNP database as known polymorphisms. For each missense SNV, functional impact was predicted using SIFT or Polyphen2 tools and identified 11 SNVs with putative damaging consequences predicted by both softwares. Twenty-five out of the 35 missense mutations were validated by Sanger sequencing and corresponded to 22 SNVs and 3 frameshift deletions. The somatic state of the validated mutations was verified with direct sequencing. Considering the whole cohort, including the discovery and the validation sets, somatic variations were found in 22% (8/37) of the PCNSL cases for *TBL1XR1*, 19% (7/37) for *PIM1*, 16% (6/37) for *ETV6*, 14% (5/37) for *IRF2BP2* and 11% (4/37) for *IRF4* and *EBF1* each (Fig 3A). Of note, 3 non-sense mutations affecting *ETV6* and *IRF2BP2* genes and 3 deletions leading to a frameshift in *TBL1XR1*, *ETV6* and *EBF1* were observed (Figure 3B). Somatic mutations on the hot spots L265P of *MYD88* and Y196 of *CD79B* were already referenced in the COSMIC database. One somatic mutation within the *PIM1* gene was also identified in this database (e.g., COSM220740) as reported in DLBCL cases [9, 14]. Four other somatic mutations identified within the *PIM1*, *ETV6* and *IRF4* genes in this study occur in the same codon as the alterations that are mainly reported in hematopoietic or lymphoid malignancies. Direct sequencing of *MYD88* and *CD79B* focused on the hot spot mutations identified in the discovery panel; the L265P mutation was found in 4/9 cases, and Y196 mutations were found in 3/9 cases. In the validation panel, 10 additional patients harbored the *MYD88* L265P mutation and 8 additional cases harbored *CD79B* Y196 mutations. Considering the whole population, *MYD88* L265P and *CD79B* Y196 mutations were identified in 38% (14/37) and 30% (11/37) of PCNSL tumors, respectively (Figure 3A), representing the most recurrently mutated genes in our series.

## DISCUSSION

The present study investigated the coding genomes of PCNSL in order to provide information on the mutational landscape of these tumors. We described an overview of the genes that are recurrently mutated in PCNSL, including (i) genes previously known to be mutated in PCNSL, such as *MYD88*, *CD79B*, *PIM1* and *TBL1XR1*; (ii) genes altered by somatic mutations in other B cell malignancies that have not yet been reported in PCNSL, such as *ETV6*, *IRF4* or *EBF1*; and (iii) genes that are altered in solid tumors, such as *IRF2BP2*. These results reveal the genetic heterogeneity of this disease and highlight the major signaling pathways that are deregulated in PCNSL.

In our series, genes coding for nuclear factor-κB (NFκB) pathway regulators (i.e., *MYD88*, *CD79B* and *TBL1XR1*) represented the most frequently altered genes. *MYD88* encodes a signaling adaptor protein that induces NFκB and JAK/STAT3 pathway activation after the stimulation of the Toll-like and IL1/IL18 receptors as well as interferon β production [17, 18]. *CD79B* encodes a B-cell receptor (BCR) subunit that is essential for BCR signaling, leading to NFκB activation [19]. We identified *MYD88* L265P and *CD79B* Y196 hot spot mutations in 38% and 30% of the PCNSL patients, respectively. We confirm and expand the results of Montesinos-Rongen et al [20, 21] who have recently investigated PCNSL for mutations in several genes involved in the BCR signaling cascade and reported a 36% (7/14) and 20% (5/25) mutation rate in *MYD88* and *CD79B*, respectively. These two hot-spot mutations have been described as oncogenic activating alterations leading to constitutive NFκB activation in DLBCL [22, 23]. Additionally, we found a significant association (*p*=0.0044, Chi-square test) between the *MYD88* L265P and *CD79B* Y196 mutations, suggesting collaborative effects of the NFκB activating pathways in PCNSL. The *TBL1XR1* gene, which encodes for a transcriptional regulator involved both in the Wnt/B catenin [24, 25] and NFκB pathways [26], was mutated in 22% of our PCNSL cohort. The *TBL1XR1* mutation rate in our series and the recurrent deletions of 3q26.32 (*TBL1XR1* locus) reported in PCNSL [7], extracerebral DLBCL [15], and acute lymphoblastic leukemia [27, 28] suggest its potential role as a tumor suppressor. Taken together, mutations in *MYD88*, *CD79B* and *TBL1XR1* affected 54% (20/37) of our cohort, suggesting that NFκB pathway deregulation is a driving mechanism in PCNSL tumorigenesis. Other genes, such as *CARD11* and *TNFAIP3*, which belong to this pathway are also reported to be mutated at lower rates in 16% and 3% of PCNSL, respectively [29].

A second set of alterations was detected in genes involved in B-cell proliferation and differentiation, such as *ETV6*, *EBF1*, *IRF4* and *ETS1*. To our knowledge these gene mutations have never been reported in PCNSL. The *ETV6* tumor suppressor gene encodes an Ets family transcriptional repressor factor required for hematopoeisis [30] and largely described as a partner of gene translocation in lymphoid and myeloid hematopoietic tumors [31]. In our series, we found *ETV6* mutations in 16% of cases, including 2 cases with non-sense mutations. In line with this, several studies have reported heterozygous and homozygous deletions of 12p13.2 corresponding to the *ETV6* locus (15% in the present series) in PCNSL [6, 7]. *IRF4*, also known as *MUM1* encoding a lymphocyte-specific transcription factor [32], and *EBF1*, encoding an activator of transcription involved in lymphoid development [33], were found to be mutated in 11% of our cohort. We also reported, in our discovery set, 2 somatic mutations affecting *ETS1*, encoding another Ets family transcription factor involved in the negative regulation of plasmocytic differentiation [34]. A variety of *ETS1* alterations, including deletions [35] or gains [36] and somatic mutations [9, 37], have been reported in B cell malignancies. Finally, 11 tumors from our 37 samples (30%) harbored one or more mutation of genes involved in B cell proliferation and differentiation, supporting the role of B lymphoid development deregulation in PCNSL tumorigenesis.

A hallmark of oncogenesis is the alteration of genes controlling the cell cycle. We and others have previously identified *CDKN2A* homozygous deletions as a frequent alteration in PCNSL [4, 6–8] with an unfavorable impact on the prognosis [8]. In the present study, we found recurrent mutations in cell cycle regulator genes such as *PIM1* [38] (7/37; 19%), *IRF2BP2* [39] (5/37; 14%) and *BTG1* [40] (2/9). *PIM1* is a proto-oncogene that encodes a serine/threonine kinase and is known to be frequently targeted by somatic hypermutation in PCNSL [41]. Of note, 6 of the 7 seven mutations identified on *PIM1* in the present study were located on the protein kinase domain. A variety of inhibitors are currently under development for PIM family proteins [42]^(Tab2)^, rendering these proteins attractive targets for therapy [5]. *IRF2BP2* encodes a zinc finger protein that interacts with partners such as *TP53* and the oncogene *IRF2. IRF2BP2* acts as a repressor of *IRF2*, leading to the inhibition of interferon responsive gene expression and *NFAT1*, which is involved in the cell cycle. Recently, a novel fusion between *IRF2BP2* and the *CDX1* homeobox gene was described in a patient suffering from a mesenchymal chondrosarcoma [43]. Intriguingly, the patient also had PCNSL; unfortunately the brain tumor tissue was not investigated.

**Figure 4:**
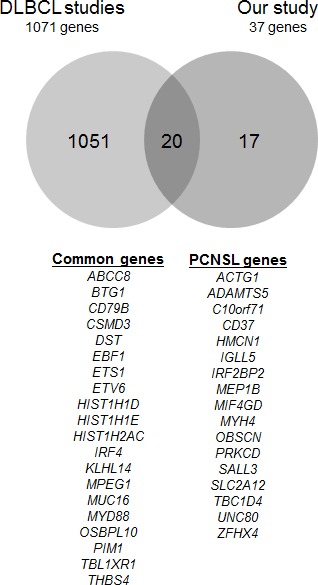
Overlaps in genes discovered in DLBCL studies and our 37 genes of interest The Venn diagram depicts the comparison between gene mutations from the five DLBCL exomes studies and the present PCNSL study. The gene lists used were as follows: Lohr et al. (Table 1 in Ref. 11, n=72 genes), Pasqualucci et al. (Table S3 and Fig. S4 in Ref. 15, n=108 validated somatic genes), Zhang et al. (Table S3 in Ref. 14, n=322 genes), Morin et al. (in Ref. 9, n=315 known and confirmed somatic genes; Table S3 in Ref. 37, n=588 known and confirmed somatic genes).

Our results revealed many similarities between genomic abnormalities of extracerebral DLBCL and PCNSL. Indeed, among the 37 genes of interest identified in this study, 20 have described mutations in DLBCL exome studies [9, 11, 14, 15, 37] (Figure 4). More specifically, mutations in the genes involved in the NFκB signaling pathway and in *PIM1*, as observed in PCNSL, are likely associated with the activated B-cell like (ABC) subtype of DLBCL. In contrast, histone-modifying genes, such as *CREBBP*, *EZH2* and *MLL2*, which are recurrently altered in the germinal center B-cell like (GCB) subtype of DLBCL [9, 14, 15], were not found in our series. These observations are in agreement with previous studies showing that the PCNSL gene expression profile is more closely related to post-GCB and ABC cells than to GCB cells [2, 44].

The present study has several limitations. Even if the small number of cases analyzed is generally acceptable given the rarity of the disease and small amount of available tissue, it provides a limited power of analysis and we likely underestimate the PCNSL gene mutations. In addition, the sequencing methods used do not investigate noncoding portions of the genome. Altogether, this could explain the relatively low overlap with a recent study of the Mayo Clinic including 10 PCNSL investigated by whole exome sequencing (O'Neill BP *et al.*, 2013, ASH Annual Meeting Abstract). Alternatively, these results could also illustrate a high molecular heterogeneity within PCNSL as observed in extracerebral DLBCL exome studies [14]. However, our results contribute to the description of the PCNSL mutational landscape and provide insights into the prominent signaling pathways that are disrupted in PCNSL tumorigenesis. Genomic similarities with the ABC subtype of extracerebral DLBCL may open the possibility for parallels in therapeutic strategies of both lymphomas. For example, lenalidomide which induces IRF4 levels decrease [45], and ibrutinib which targets B-cell receptor signaling (Wilson WH *et al.*, 2012, ASH Annual Meeting Abstract) have shown promising results in extracerebral ABC-DLBCL. In this setting, they might also be attractive therapeutic strategies for PCNSL.

## METHODS

### PCNSL sample selection and patient characteristics

Thirty-seven PCNSL patients were selected for the present study. All tumors were classified as CD20+ DLBCL according to the WHO classification and demonstrated to contain at least 90% tumor cells based on morphology and immunohistochemistry. All the patients were newly diagnosed and immunocompetent. The participants provided written consent for sample collection and genetic analysis. This study was approved by the local ethical committee (CPPRB Pitié-Salpêtrière). Based on the high quality and sufficient levels of DNA, nine paired frozen tumor and blood tissues were selected to constitute the discovery set investigated by whole exome sequencing, and 28 tumor samples constituted the validation set investigated by direct sequencing. The sex ratio was 1.18 (male/female) and the median age at diagnosis was 61 years, ranging from 17 to 83.

### Isolation and quality assessment of DNA

Tumor DNA from 34 cryopreserved and 3 FFPE samples was extracted using the QIAamp DNA Mini Kit (Qiagen) and iPrep™ ChargeSwitch® Forensic Kit (Life Technologies), respectively, according to the manufacturer's instructions. A conventional saline method was used for the extraction of germline DNA from the blood samples. DNA was quantified using a NanoDrop spectrophotometer, and the quality was assessed on a 1% agarose gel.

### Whole exome sequencing

Whole exome sequencing was possible for PCNSL patients with available paired frozen tumor and blood samples and with a minimal amount of 5 μg of tumor and germline DNA. Genomic DNA capture was performed using biotinylated oligonucleotides probes library (Human All Exon v2 – 46 Mb, Agilent) according to Agilent in-solution enrichment methodology (SureSelect Human All Exon Kits Version 2, Agilent). Sequence capture, enrichment and elution were performed according to manufacturer's instructions and protocols (SureSelect, Agilent). Massively parallel sequencing was realized on an Illumina GAIIX as paired-end 75 b reads.

### Mapping and variant calling

Mapping of high-quality paired-end sequenced reads onto the GRCh37 build of the human reference genome was performed by Integragen using the Illumina ELAND 2 software tool. Raw alignments were first filtered for both low-quality mapped reads and assumed PCR duplicates with the SAMtools view (-q 20) and the Picard MarkDuplicates utilities, respectively [46]. The resulting filtered BAM files were subsequently confined to the genomic coordinates delineating the Agilent SureSelect 50-Mb probes using the intersectBed command of the BEDtools suite [47]. A commonly used combination of SAMtools mpileup and BCFtools view was then applied to the latter bounded alignments in order to call single nucleotide variations (SNVs) as well as short insertions and deletions (indels) within the targeted genomic regions. Mapping and coverage summary statistics were additionally obtained by an in-house post-processing of SAMtools idxstats and mpileup outputs.

### Annotating called variants

Variant annotation was performed with the unpublished Genomic and Functional Annotation Pipeline (GFAP) software, developed and routinely used at Institut Curie (http://gfap.curie.fr/). Briefly, GFAP consists of a set of tools that automatically: (i) retrieve and store suitable information from public variant databases such as 1000-genomes [48], dbSNP [49] or COSMIC [50, 51], (ii) match submitted variants against built-in databases and annotate them with respect to their genomic localization, (iii) assign an integrated functional impact prediction to non-synonymous variants (including stop-gains and losses) using dbNSFP database [12, 13] which compiles several tools such as SIFT [52] or Polyphen2 [53].

### Validation set

Samples were selected based on the availability of tumor DNA. The validation set was investigated for known hotspot mutations by Sanger sequencing and for all exons of highly mutated genes by pyrosequencing. The tumor DNA was amplified using the primers listed in Supplementary Table 3. The amplification conditions were 94°C for 3 min followed by 45 cycles of 94°Cx15 sec, 60°Cx45 sec and 72°Cx1 min, with a final step at 72°C for 8 min. Exon 13 of *TBL1XR1* was amplified using Touch Down PCR with a gradient from 62 to 55°C during 6 cycles followed by 30 cycles at 55°C for primer annealing. The PCR products were purified according to the Agencourt® AMPure® XP PCR purification protocol (Beckman Coulter) with the Biomek® 3000 Automation Workstation.

### Sanger sequencing

Sequencing reactions were performed in both orientations using the Big-Dye® Terminator Cycle Sequencing Ready Reaction (Perkin Elmer). The extension products were purified with the Agencourt® CleanSEQ® protocol according to the manufacturer's instructions (Beckman Coulter). The purified sequences were analyzed on an ABI Prism 3730 DNA Analyzer (Applied Biosystems). The forward and reverse sequences were visualized using Chromas Lite software.

### Pyrosequencing

The universal tailed amplicon resequencing approach (454 Sequencing Technology, Roche) was used for coding exons sequencing. This system employs a second PCR, aiming MID (multiplex identifier) and 454 adaptors incorporation, an emulsion PCR according to the emPCR Amplification Method Manual Lib-A protocol (GS Junior Titanium Series, Roche), enrichment and pyrosequencing according to the Sequencing Method Manual (Roche). Sequences analysis was performed using CLC Genomics Workbench software.

## SUPPLEMENTARY FIGURES AND TABLES




