# Supplementary material for: Mutational analysis of primary central nervous system lymphoma
Source: Oncotarget. 2014 Jun 8;5(13):5065–75. doi: 10.18632/oncotarget.2080 (PMC4148122; doi:10.18632/oncotarget.2080)
Supplement: Supplementary file 2 [file oncotarget-05-5065-s002.pdf]

Mutational analysis of primary central nervous system lymphoma

Supplementary Material

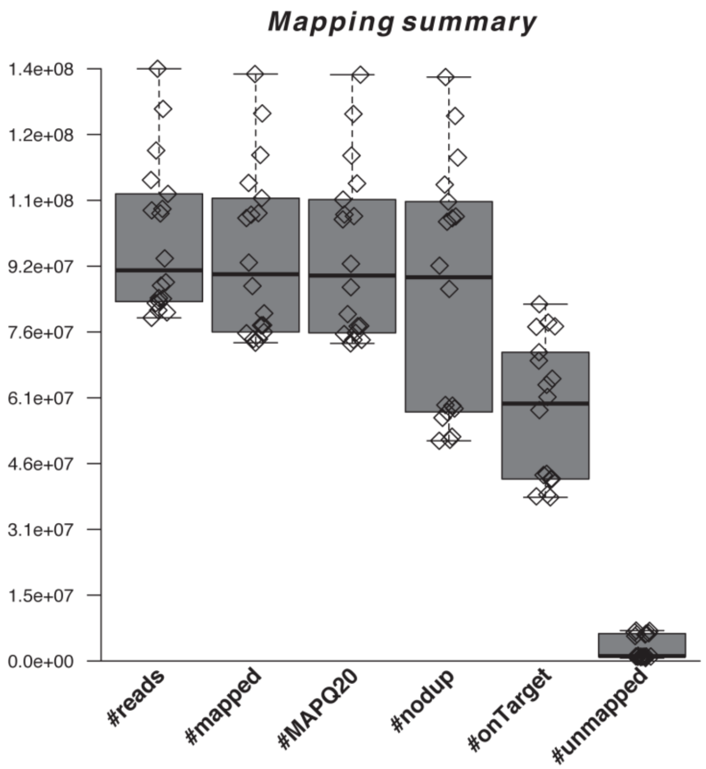

Supplementary Figure 1: Mapping summary statistics of exome sequencing experiments. Each dot represents one the 18 sequenced samples. #reads: total number of sequenced paired-reads; #(un)mapped: reads (un)mapped onto the GRCh37 build of the human reference genome; #MAPQ20: subset of #mapped with a Phred-scaled mapping quality score greater or equal to 20; #nodup: subset of #MAPQ20 without potential PCR-derived duplicates; #onTarget: subset of #nodup overlapping Agilent SureSelect 50Mb probes.

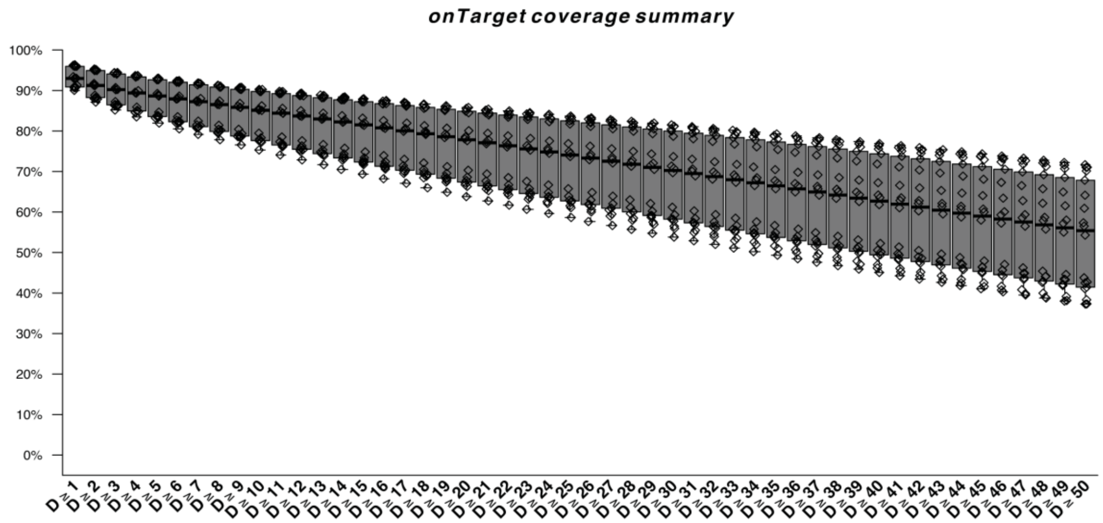

Supplementary Figure 2: Coverage summary statistics of exome sequencing experiments. Each dot represents one the 18 sequenced samples. X-axis represents the depth of coverage. Y-axis represents the relative fraction of the human reference exome defined by the Agilent SureSelect 50Mb probes. Only high-quality mapped reads with no duplicates are considered.

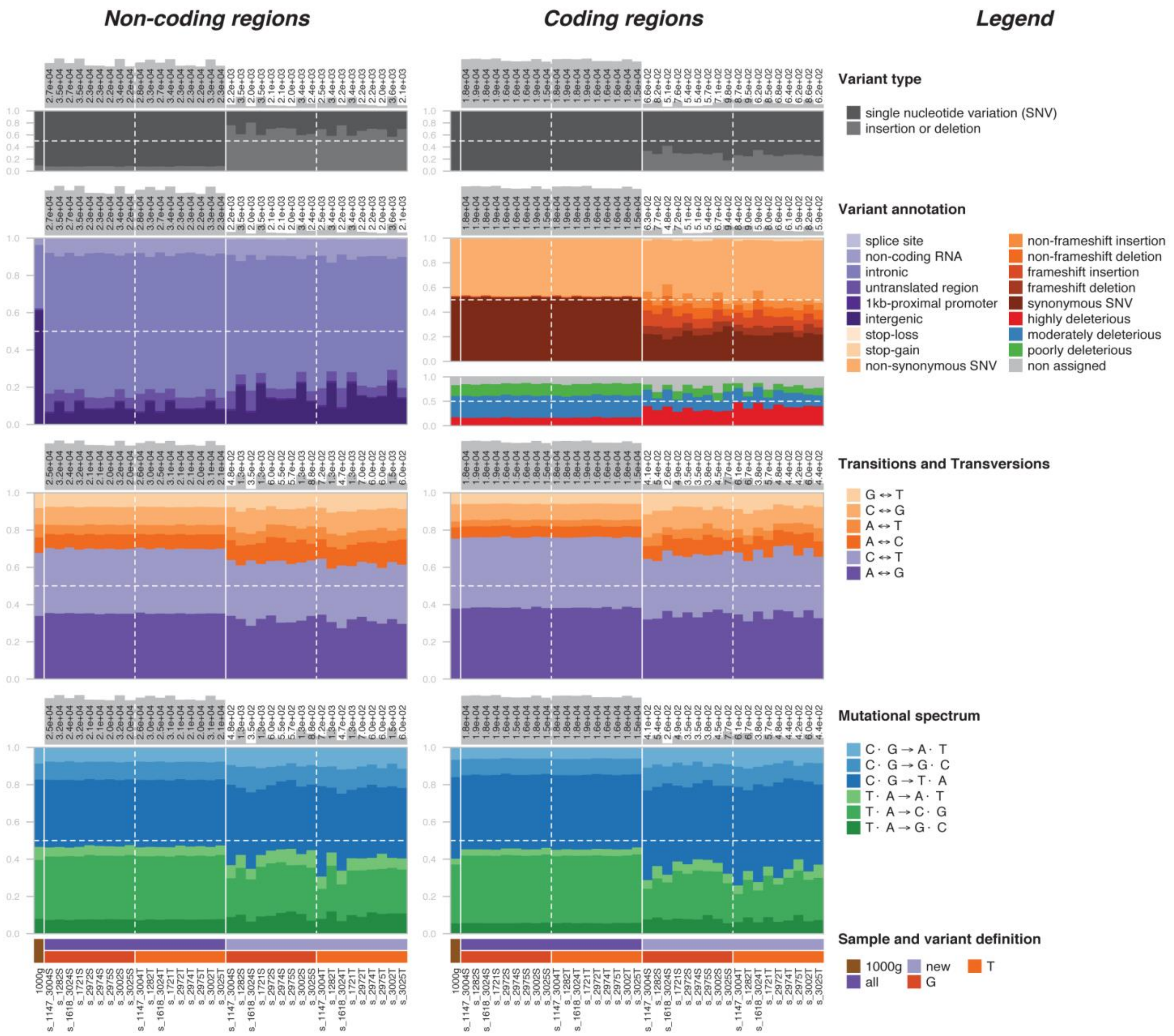

Supplementary Figure 3: Mutational profile of PCNSL samples investigated by whole exome sequencing. Nine paired blood and tumor DNA were processed using high throughput exome sequencing. Patterns of the 18 samples analyzed are illustrated for coding regions. In X axis, from the left to the right, data from 1000 genome (1000g), germline (G) and tumor (T) samples with a discrimination between all variants which pass quality criteria (all) and only variants not described as known polymorphisms (new). The vertical axis represents the proportion of each variant type.

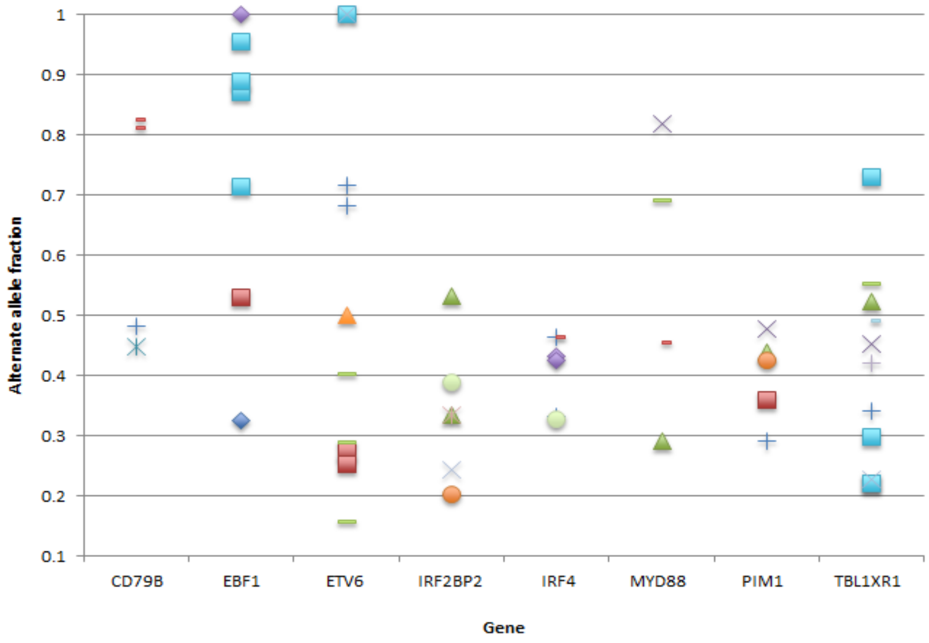

Supplementary Figure 4: Distribution of variant allelic frequencies. Average allelic fractions are represented for all validated somatic mutations found within the 8 genes of interest in the discovery and the validation sets. This information was not available for *MYD88* and *CD79B* hot spots found by Sanger sequencing. X-axis represents the 8 genes of interest. Y-axis represents allelic ratios - i.e AD/DP with AD the number of reads consistent with the alternate allele and DP the total of reads. Patients are indicated with different symbols or colors.
